# A new way to maintain CD8^+^ T-cell quiescence: interaction between CD8α and PILRα

**DOI:** 10.1038/s41392-022-01094-9

**Published:** 2022-07-12

**Authors:** Ke Jin, Bowen Wu

**Affiliations:** 1 Laboratory of Human Diseases and Immunotherapies, West China Hospital, Sichuan University, 610041 Chengdu, China; 2 Institute of Immunology and Inflammation, Frontiers Science Center for Disease-related Molecular Network, West China Hospital, Sichuan University, 610041 Chengdu, China; 3 Department of Medicine, Mayo Clinic College of Medicine and Science, Rochester, MN 55905 USA

**Keywords:** Lymphocytes, Molecular medicine

Recently, a paper published in *Science* by Zheng et al. reported that CD8α was crucial to physiologically maintain the quiescent state of CD8^+^ T cells in peripheral lymphoid organs. Paired immunoglobulin-like type 2 receptor alpha (PILRα) was recognized as the ligand for CD8α and blockade of PILRα-CD8α interaction would disrupt CD8^+^ T-cell quiescence.^1^

Due to the superior cytotoxic and killing ability to eliminate invading pathogens, infected cells, and tumor cells in human body, CD8^+^ T cells have gained long-term attention from immunologists. The balance between survival and death of CD8^+^ T cells in the T-cell pool is essential to sustain their biological functions and to keep the homeostasis of body. When exposed to major histocompatibility complex (MHC) class-I recognized antigens, CD8^+^ T cells exit quiescence to initiate clonal expansion and differentiate into cytolytic effector T cells quickly. After the clearance of pathogens or antigens, most effector T cells die, with a small fraction surviving and becoming the long-lasting memory T cells.^2^ CD8^+^ T-cell death is generally triggered by programmed cell death (PCD), such as activation-induced cell death (AICD) which is mediated by Fas–FasL interaction. Another PCD of CD8^+^ T cell usually occur in T-cell “exhaustion”, which is triggered by the upregulation of numerous inhibitory immune checkpoint receptors.^2^ The most typical example is programmed cell death 1 (PD-1) and its interaction with PD-1 ligand (PD-L1) will lead to T-cell exhaustion and the subsequent T-cell death.^2^

**Fig. 1:**
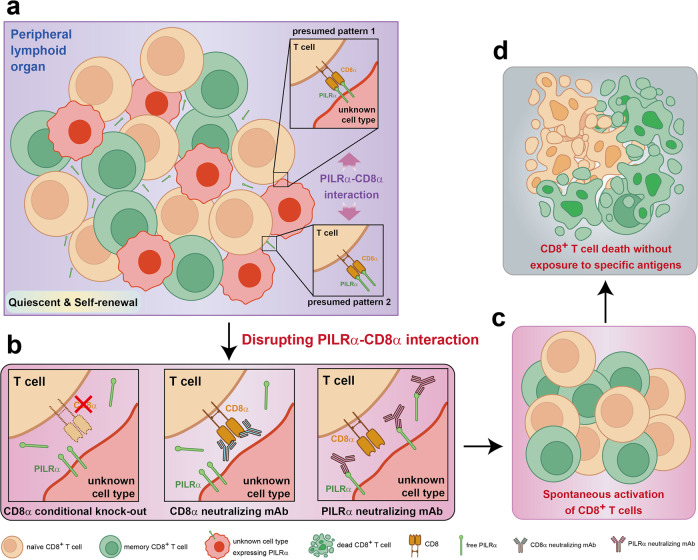
CD8α-PILRα interaction maintains the physiologically quiescent state of naive and memory CD8^+^ T cells in peripheral lymphoid organs. **a** In the steady-state, CD8 molecule expressed by naive and memory CD8^+^ T cells was occupied by PILRα (free or expressed by some cell types?) in the absence of antigen exposure to avoid unnecessary activation and AICD of CD8^+^ T cells. **b**–**d** Disruption of CD8α-PILRα interaction by conditional deletion of CD8 molecule or treatment with CD8α or PILRα neutralizing mAbs (**b**) leads to spontaneous activation of naive and memory CD8^+^ T cells without exposure to specific antigens (**c**) and the subsequent CD8^+^ T cell death (**d**)

In this study, Zheng et al. identified a novel PCD of CD8^+^ T cell mediated by CD8α deletion. In CD8α knock-out T cells, cleaved caspase-3 and Fas expressions were elevated, which represented the activation of canonical PCD pathways. Actually, this is a subversive discovery. CD8 generally functions in the form of either a heterodimer composed of one alpha chain and one beta chain or as a homodimer composed of two alpha chains. The CD8 serves as a coreceptor with the T-cell receptor (TCR) on T cells for their activation and clonal expansion to recognize antigens presented by MHC class-I molecules on antigen-presenting cells (APCs). Therefore, few people will consider that CD8α is related to T-cell quiescence. However, Zheng et al. found that without specific antigen stimulation, CD8^+^ T cells acquired a loss-of-quiescence phenotype upon deletion of CD8α. In this study, they generated tamoxifen-inducible CD8 conditional knock-out (*Cd8a*^fl/fl^*Cre*-*ER*^T2^, same as *Cre*^+/+^*Cd8a*^loxp/loxp^ in the *Science* paper) in transgenic mice. Hereafter, purified CD8^+^ T cells (CD45.2) from *Cd8a*^loxp/loxp^*Cre*^+/+^ and *Cd8a*^+/+^*Cre*^+/+^ (control) mice were adoptively transferred into wild-type mice (CD45.1) respectively, followed by tamoxifen treatment to conditionally knockout CD8α in peripheral CD8^+^ T cells (CD45.2) without affecting thymic development. Thirty-four and thirty-six days after tamoxifen treatment, survival of transferred *Cd8a*^loxp/loxp^*Cre*^+/+^ CD8^+^ T cells was 30–40% lower compared to control CD8^+^ T cells, in both naive and memory population. Meanwhile, T-cell activation markers CD69 and Fas were upregulated upon CD8α deletion. They also found that quiescence loss induced by CD8α deletion was not caused by TCR triggering, because CD5 which transmitted a tuning signal for TCR and Nur77, an immediate-early protein downstream of TCR, were both decreased in memory and naive CD8α^−/−^ cells. Disruption of CD8^+^ T-cell quiescence was also confirmed in thymectomized mice by utilizing anti-mouse CD8α monoclonal antibody (mAb, clone 3D9) generated by the authors in this study (Fig. 1).

Given that CD8α helps to activate CD8^+^ T cells, there may be a ligand with the ability to interact with CD8α to block the activation signal transmission, maintaining the quiescent state of CD8^+^ T cells. To uncover this potential ligand, Zheng et al. performed a high-throughput screening. On the surface of 293T cells, 6000 human transmembrane proteins were individually displayed. Then they tested the binding potential of human CD8α to these proteins. As a result, PILRα was identified as a binding ligand for CD8α. To evaluate the role of PILRα in maintaining the quiescent state of CD8^+^ T cell, the authors generated another specific antibody against mouse PILRα (clone 9B12) which could block PILRα−CD8α interaction. By 9B12 treatment, blockade of PILRα-CD8α interaction increased CD8^+^ T-cell activation with interferon-γ (IFN-γ) and CD69 upregulation. Treatment of 9B12 impaired the survival and disrupted quiescence of naive and memory CD8^+^ T cell as well in their study. As a transmembrane protein, PILRα is broadly expressed on myeloid cells, including dendritic cells, macrophages, and neutrophils.^1^ For neutrophils, PILRα negatively regulates neutrophil infiltration during inflammation, and *Pilra* knock-out mice have increased neutrophil recruitment to inflammatory sites and are highly susceptible to endotoxin shock.^3^ It implies that PILRα exerts critical functions in innate immunity. This study may link functions of PILRα in innate immunity and adaptive immunity. An intriguing question is that since PILRα is dominantly expressed on innate immune cells, how do innate immune cells influence the quiescent state of CD8^+^ T cells under physiological and/or pathological conditions? Do innate immune cells indeed regulate CD8^+^ T-cell quiescence by PILRα-CD8α interaction? On the other hand, the underlying mechanism that controls the interaction between PILRα and CD8α is still unknown. For instance, how does PILRα unbind CD8α when CD8^+^ T cells are exposed to antigens for activation and clonal expansion? This study provides us with cues and ideas to figure out these questions.

Conclusively, understanding the related programs regulating T-cell quiescence is critical for developing novel approaches to modulate protective and pathological T-cell responses in human diseases.^4^ T-cell replenishment relies more on homeostatic self-renewal of naive T cells instead of thymic activity. In addition, the thymus undergoes involution during childhood and adolescence, resulting in the reduction of thymic epithelial cells and thymocytes and disruption of the tissue architecture.^5^ Therefore, naive T-cell expansion is vital for T-cell replenishment. The current study identifies that PILRα-CD8α interaction can maintain the quiescence of both naive and memory CD8^+^ T cells in peripheral lymphoid organs independent of thymic context, avoiding activation by stimulation. Therefore, PILRα-CD8α interaction may become a new avenue for CD8^+^ T-cell replenishment without AICD. Besides, promoting PILRα-CD8α interaction is also a promising strategy to sustain T-cell homeostasis.
